# Spesolimab for treatment of generalized pustular psoriasis of pregnancy

**DOI:** 10.1002/pmf2.70196

**Published:** 2025-12-08

**Authors:** Jason Bunn, Christine Henricks, Aaminah Azhar, Cristina Thomas, Edward Hadeler, Alexandra Smith, R. Nicholas Burns, Jarad Levin, Marvin Williams, Hugh Nadeau

**Affiliations:** ^1^ Division of Maternal‐Fetal Medicine Department of Obstetrics and Gynecology University of Oklahoma Medical Center Oklahoma City Oklahoma USA; ^2^ Division of Maternal‐Fetal Medicine Department of Obstetrics and Gynecology University of Texas Southwestern Medical Center and Parkland Hospital Dallas Texas USA; ^3^ Department of Dermatology University of Oklahoma Medical Center Oklahoma City Oklahoma USA; ^4^ Department of Dermatology University of Texas Southwestern Medical Center and Parkland Hospital Dallas Texas USA; ^5^ College of Osteopathic Medicine Oklahoma State University Oklahoma City Oklahoma USA

**Keywords:** generalized pustular psoriasis, IL‐36 receptor, impetigo herpetiformis, pregnancy dermatoses, pustular dermatitis, spesolimab, spesolimab pregnancy

## Abstract

**Introduction:**

Generalized pustular psoriasis (GPP) is a severe, desquamating skin disorder associated with pregnancy that presents as fever, fatigue, malaise and sterile pustules. It is associated with profound maternal morbidity, maternal mortality, placental insufficiency and stillbirth. Treatment often includes glucocorticoids that are continued until delivery. Monoclonal antibody spesolimab was approved for treatment of GPP in 2022 but there is a paucity of pregnancy data.

**Case:**

A 39‐year‐old multiparous African American patient with chronic hypertension presented at 19 weeks gestational age with scaling rash, which progressed to blistering and sloughing several days later. She required burn intensive care. Her disease was refractory to high‐dose glucocorticoids, cyclosporine and TNF‐α inhibitors. Ongoing corticosteroid usage exacerbated her hypertension and resulted in hyperglycemia. Spesolimab was administered, which resulted in a rapid resolution of GPP within days of administration, and this was maintained throughout her pregnancy. There were no sustained maternal or fetal effects and she delivered a healthy term neonate.

**Conclusions:**

Treatment with spesolimab rapidly improved symptoms of GPP refractory to other immunomodulators. This is the first published use of spesolimab in a pregnant patient in the United States. We suggest that spesolimab may offer rapid, safe relief of severe immunologic disease in pregnancy, though further study is required.

## 1 INTRODUCTION

Generalized pustular psoriasis (GPP) is a severe, desquamating skin disorder associated with pregnancy and may result in placental insufficiency, severe maternal morbidity and maternal mortality [1, 2, 3]. Corticosteroids are the mainstay of treatment though alternatives include cyclosporine and TNF‐α inhibitors [1, 2, 4]. Spesolimab is an IL‐36 receptor (IL‐36R) antagonist approved for GPP treatment, specifically, though it is unstudied in pregnancy. We present a 39‐year‐old multiparous patient whose GPP significantly improved with intravenous spesolimab without adverse maternal or fetal effects. We suggest this medication may offer safe, rapid relief in persons for whom pregnancy limits therapeutic options.

## 2 MATERIALS AND METHODS

The patient signed written consent for the publication of her care and images and for the sharing of data. This project did not meet the criteria for human subjects research.

### 2.1 Case

The patient is a 39‐year‐old African American gravida eight, para six, abortus one whose medical history was complicated by advanced maternal age, grand multiparity, obesity with body mass index (BMI, kg/m^2^) of 33, chronic hypertension, history of preeclampsia in a prior pregnancy, history of spontaneous preterm birth at 29 weeks gestational age (GA) and a history of two prior cesarean deliveries (one classical). She was started on labetalol 100 mg twice daily soon after her new obstetric visit.

Approximately 1 week later, the patient presented to an emergency room with complaints of itching, abdominal pain and decreased fetal movement. She had eaten shellfish the night prior. Her rash was described as scaling and confluent across her entire trunk and extremities. Over the following week, she presented several times to the emergency room and once to her obstetrician for a worsening rash, which did not improve despite oral methylprednisolone and antihistamines.

At her fourth emergency room presentation at 19 weeks GA she reported further worsening of her rash, severe pain and decreased fetal movement. Her vital signs were significant for a blood pressure of 106/90 mmHg, heart rate of 109 beats/min and temperature of 36.6 degrees. Her exam was significant for diffuse blistering and skin sloughing though sparing of mucous membranes. The patient was promptly transferred to an inpatient maternal‐fetal medicine service.

Her vital signs and exam were unchanged following transfer; her labs were significant for a leukocytosis of 23.7 × 10^3^/µL, potassium 3.1 mmol/L, creatinine 0.74 mg/dL, lactate 3.2 mmol/L and ionized calcium 1.08 mmol/L. Blood and urine cultures were negative. Her routine obstetric serologies were negative, including syphilis and HIV. Labetalol was discontinued on admission. The patient received IV hydration and IV hydromorphone for pain and a Foley catheter was placed.

**FIGURE 1:**
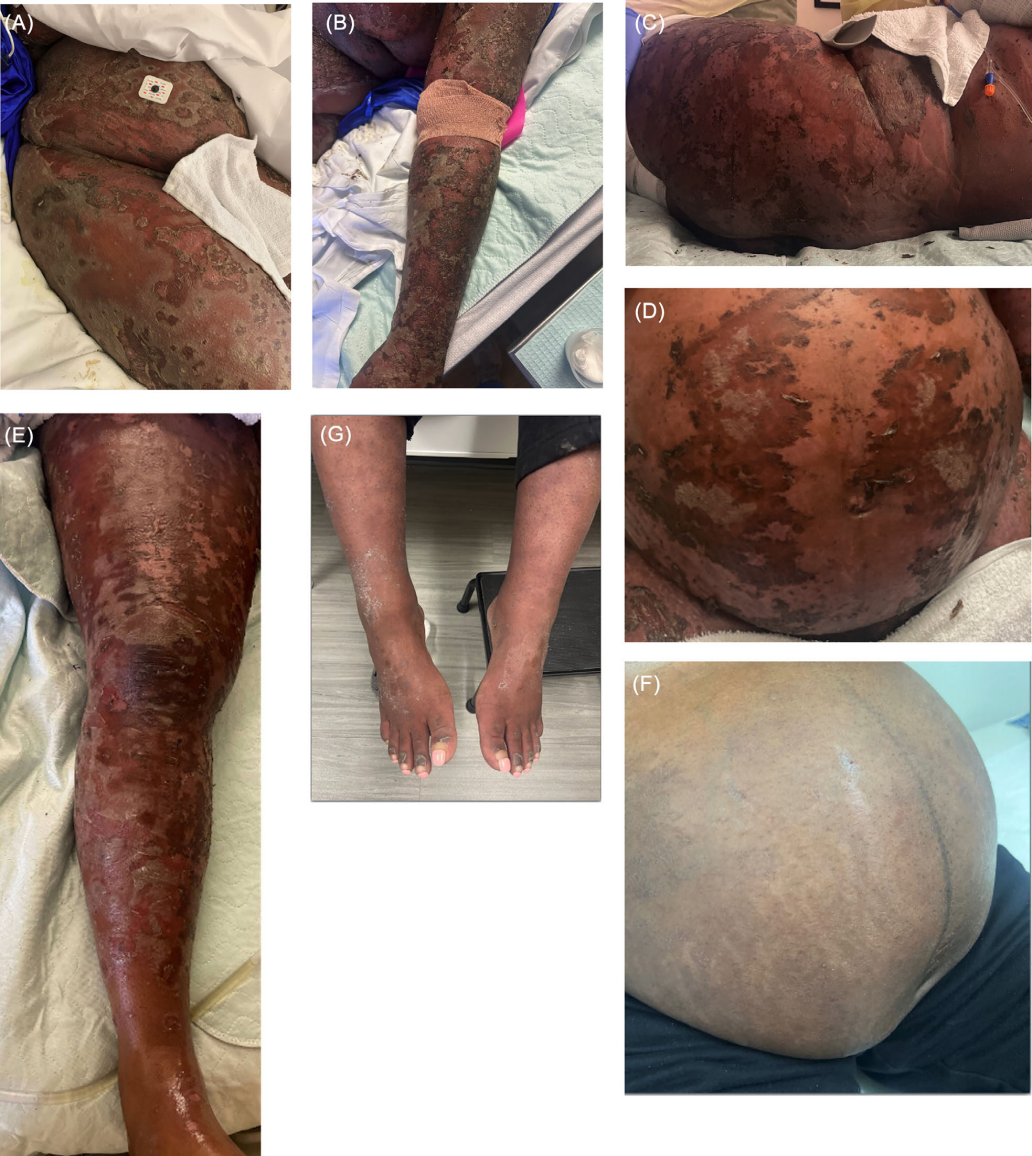
Admission revealed rapidly progressive polymorphous, desquamative, erythematous plaques on (A,B) upper extremities, (C) flank, (D) abdomen, (E) lower extremity. Resolution of active disease at 30 weeks GA, 2 months after initial infusion on (F) abdomen and (G) lower extremities. GA, gestational age.

A dermatology consultation further defined her skin exam: annular plaques with sheets of pustules and superficial desquamation on the scalp, trunk and bilateral extremities without mucous membrane involvement (Figure 1). Punch biopsy of the skin demonstrated intraepidermal neutrophilic pustules which supported a diagnosis of pustular psoriasis of pregnancy. Direct immunofluorescence testing was negative. She was initiated on IV methylprednisolone 0.5 mg/kg twice daily, topical corticosteroids and empiric cephalexin. Her skin findings only continued to worsen and she was transferred for burn intensive care at 19 weeks 2 days GA.

She was discharged at 27 weeks 1 day GA. She had received 10 days of IV cefazolin, 25 days of IV cyclosporine, a prolonged steroid course (tapered at discharge) and two doses of infliximab. The patient had been counseled extensively by maternal‐fetal medicine who reviewed all published and manufacturer data on IV spesolimab and two doses were administered. Her skin exam markedly improved within 1 week of her first spesolimab infusion and discharge assessment revealed few remaining pustules with diffuse xerosis. Her hospital course was otherwise complicated by severe chronic hypertension necessitating a four‐drug regimen, corticosteroid‐induced hyperglycemia and profound deconditioning.

Her subsequent care was managed by maternal‐fetal medicine and dermatology. Subcutaneous spesolimab 300 mg was continued every 4 weeks. Her insulin was discontinued following her steroid taper but her hypertension persisted. The fetus underwent detailed ultrasounds at 23 and 27 weeks, which both demonstrated appropriate growth and normal anatomy. She underwent growth ultrasounds every 4 weeks and weekly biophysical profiles starting at 32 weeks GA.

She presented for her scheduled repeat cesarean delivery at 37 weeks GA. The procedure was uncomplicated; the estimated blood loss was 765 mL. She delivered a female neonate weighing 2765 g with APGAR scores of 8 and 9 at 1 and 5 min, respectively. The arterial cord gas revealed a pH of 7.32 with a base excess of −0.3 mmol/L. Her postpartum course was complicated by severe postoperative pain, poor mobility and insulin therapy following stress‐dose steroid administration. Her baby had a reassuring initial evaluation and uncomplicated hospital stay and was discharged with her mother on postoperative day 4.

The patient had immediate follow‐up with her primary provider and was evaluated by maternal‐fetal medicine roughly 2 weeks after her cesarean. A computed tomography (CT) scan revealed a lower anterior midline bowel‐containing hernia and focal epiploic fat inflammation suspicious for epiploic appendagitis. She sought follow‐up with dermatology, maternal‐fetal medicine and general surgery. Her follow‐up exams revealed resolving mild xerosis on her trunk and extremities and mild facial acne vulgaris, similar to exams before delivery (Figure 1).

## 3 DISCUSSION

GPP of pregnancy (formerly known as impetigo herpetiformis) often manifests in the third trimester, resolves with delivery, and is associated with stress, pregnancy, hypocalcemia, corticosteroid withdrawal and beta blockade [1, 2]. Signs and symptoms include fatigue, malaise, leukocytosis and sterile pustules in intertriginous areas that spread and desquamate centrifugally. Maternal mortality may be as high as 16% as a consequence of septic shock or cardiopulmonary failure [4]. Fetal outcomes such as placental insufficiency or stillbirth have been described [1, 2, 3]. The first GPP‐specific treatment, spesolimab, was recently approved and there is thus a significant unmet therapeutic need for it [5].

Our patient's atypically early presentation prompted the initiation of first‐line corticosteroids followed by prolonged cyclosporine treatment and multiple infusions of IV infliximab without improvement. Prolonged steroid dosing resulted in a profound hyperglycemia necessitating greater than 100 units of NPH insulin each morning and it may have contributed to her chronic, severe hypertension. Persistent skin findings perpetuated ongoing pain, immobility and deconditioning. Spesolimab resulted in rapid improvement of her symptoms and this was maintained with subcutaneous dosing. Despite few data and a general hesitancy to prescribe novel therapeutics in pregnancy, treatment with spesolimab prevented the prolongation of a painful, debilitating skin condition and truncated its myriad downstream systemic effects for both mother and fetus. Glucocorticoids are often continued through delivery if severe disease is unimproved [2]. Hyperglycemia, superinfection, immobility, severe hypertension, growth restriction and preterm rupture of membranes all hasten adverse pregnancy outcomes and should encourage physicians to seek appropriate alternatives to glucocorticoids when indicated.

Spesolimab was approved as a first‐in‐class medication for GPP in 2022 [6]. It is an IL‐36 receptor antagonist that inhibits downstream proinflammatory, profibrotic pathways [5, 7]. Molecular studies confirm the downregulation of differentially expressed genes compared to nonlesional controls [7]. Its degradation pathway results in small amino acids and peptides, likely similar to endogenous immunoglobulin G [6]. Randomized trial data note a high rate of rapid response of skin lesions compared to placebo and overall encouraging quality‐of‐life data for those treated [4, 5].

Despite these positive findings, there is a paucity of data regarding active treatment in pregnancy. Yang et al. reported a similar case in China of biopsy‐proven GPP at 15 weeks GA treated with spesolimab that spared a patient significant morbidity throughout the majority of her gestation without adverse effects [3]. A similar case in the United States with antepartum spesolimab therapy has not been reported. Safety concerns include an increased risk of infection, which was observed in a higher‐than‐expected proportion of those receiving treatment in randomized studies. These commonly included urinary tract infections and influenza. A total of 12% of patients who received spesolimab reported serious adverse events and 46% were found to have anti‐drug antibodies [4, 5]. There are no head‐to‐head trials with immunomodulating alternatives. This disease's both episodic and severe manifestations render prolonged treatment with a placebo unethical [4, 5]. Robust data, particularly in pregnancy, are not anticipated.

Incomplete data often complicate obstetricians’ counseling. These deficiencies are always weighed against maternal‐fetal morbidity, which is often the most significant risk [8]. Many immunomodulators may be used in pregnancy and previous concerns regarding immunosuppression were likely overstated [8]. A single report of a novel immunomodulator without identifiable harm hardly proves safety or efficacy. Indeed, minor risks in a study population may pose considerable, undue risk in pregnancy. Concerns regarding spesolimab should be weighed against confirmed adverse effects of prolonged corticosteroids or active maternal disease until rigorous data solidify its use in pregnancy.

## 4 CONCLUSION

We present the first published case of antepartum GPP successfully treated with spesolimab in the United States. The first‐in‐class monoclonal antibody spesolimab resulted in rapid improvement in a patient refractory to corticosteroids, cyclosporine and infliximab without adverse effects. Further study is necessary to determine if IL‐36R antagonists represent a new class of therapeutics that may result in rapid, safe resolution of serious immunologic disease in pregnancy.

## CONFLICT OF INTEREST STATEMENT

The authors declare no conflicts of interest.

## FUNDING INFORMATION

The authors received no specific funding for this work.

## ETHICS STATEMENT

This project did not meet the criteria for human subjects research (University of Oklahoma IRB #18426).

## Data Availability

Data sharing is not applicable to this article as no datasets were generated or analyzed during the current study.
